# Workplace anxiety leading to job burnout among young and middle-aged university lecturers: mechanism and mitigation strategies

**DOI:** 10.3389/fpsyg.2024.1495718

**Published:** 2025-01-06

**Authors:** Kun Zhou, Jin Wang

**Affiliations:** ^1^College of Humanities and Social Science, Hebei Agricultural University, Baoding, China; ^2^College of Landscape Architecture and Tourism, Hebei Agricultural University, Baoding, China

**Keywords:** young and middle-aged university lecturers, workplace anxiety, job burnout, sense of self-efficacy, psychological influence mechanism

## Abstract

**Introduction:**

A growing group of young and middle-aged lecturers in universities, during a time of increasing career aspirations, not only bears the significant responsibility of teaching but also serves as the backbone for promoting the high-quality development of higher education in our country. Simultaneously, they are experiencing varying degrees of workplace anxiety.

**Methods:**

This paper adopts the mixed-methods of questionnaire analysis and semi-structured interview. Firstly, it presents a questionnaire designed to assess perceptions of workplace anxiety, job performance, and job burnout. Secondly, data were collected from 506 young and middle-aged lecturers across 10 colleges and universities in the four provinces of Chongqing, Anhui, Beijing, and Hebei in China through online questionnaires. A total of 449 valid questionnaires and 56 interview transcripts were obtained. Subsequently, SPSSAU online data analysis software was employed to test the hypotheses derived from the questionnaire data using confirmatory factor analysis, correlation analysis, and Bootstrap sampling analysis. Additionally, qualitative analysis was conducted in conjunction with the interview transcripts.

**Results:**

The results indicate that, on one hand, there is a significant positive relationship between the perception of workplace anxiety and the career growth expectations of young and middle-aged university lecturers. This is evidenced by a negative correlation with their perception of job performance and a positive correlation with their perception of competitive pressure. On the other hand, self-efficacy plays a moderating role in alleviating workplace anxiety and burnout among young and middle-aged university lecturers. As their sense of self-efficacy increases, their feelings of workplace anxiety diminish.

**Conclusion:**

The increasing competition among peers has posed significant challenges to the physical and mental health of young and middle-aged university lecturers. Reforming competitive mechanisms and establishing a career support system are essential, evidence-based strategies for alleviating workplace anxiety and enhancing professional efficacy. This article explores the relationship between workplace anxiety and job burnout among young and middle-aged university lecturers, while also proposing strategies for mitigation. These insights are crucial for promoting the career development of university lecturers and safeguarding their physical and mental well-being.

## 1 Introduction

Young and middle-aged university lecturers typically possess advanced degrees and are actively engaged in the pursuit of academic and professional advancement. They demonstrate a strong sense of responsibility and career ambition, positioning themselves as a crucial force in the advancement of academic disciplines and the reform of teaching practices. Additionally, they serve as the backbone of societal contributions. However, the pressure to achieve academic progress can lead to heightened anxiety (Li, 2022). Bao Wei, a researcher at the School of Education at Peking University, conducted a study on the impact of workplace anxiety on the health of university lecturers. In the research report, he specifies, “The physiological health of young and middle-aged lecturers in universities must be prioritized, and the phenomenon of ‘midlife crisis’ must be taken seriously.” In recent years, there have been distressing incidents of depression, sudden death, and illness among these educators, resulting in significant losses for the country, society, and their families.

The internalization of career growth pathways and mechanisms for lecturers has become a prevalent issue in education sector (Xu, 2021). Young and middle-aged university lecturers are experiencing the adverse effects of the prevalent policy of “either promotion or departure.” They frequently grapple with the dual pressures of conforming to external expectations and establishing their own identities, often leading to a state of workplace anxiety (Tian and Jiang, 2022). The career promotion path, which is predicated on the expectation of long-term employment, is intensifying the conflict between university management and faculty development. This system is generating heightened psychological pressure on the “Green Pepper” group and indirectly contributing to the entrenched “Title-centric” and “Paper-oriented” phenomena.

Within the Chinese higher education system, green pepper refers to young lecturers who are not only a key component of the Chinese higher education talent pool but also a crucial factor in enhancing the quality of teaching. Consequently, their mental health is essential for China to develop into a powerful nation in education. However, various pressures, including academic promotion, scientific research, teaching assessments, and family responsibilities, are emerging as significant factors that negatively impact their mental well-being. In addition, the prolonged absence of a sense of achievement in scientific research, teaching, social services, and professional title evaluations has contributed to a rising trend of “opt-out” among university faculty. This phenomenon manifests as mechanical engagement in teaching, neglect of research responsibilities, and a general lethargy in daily life, all of which are key indicators of teacher burnout (Wang and Che, 2023) while labor alienation has emerged as a significant factor contributing to this burnout. Consequently, workplace anxiety and burnout have become the two primary obstacles impeding the professional growth of young and mid-aged university lecturers (Li and Bu, 2023).

Job burnout has emerged as a prominent issue in the study of teachers’ mental health. However, existing research indicates that the relationship between teacher efficacy and job burnout varies significantly across different age groups (Chen and Tang, 2024). Self-efficacy is either positively (Brouwers et al., 2011) or negatively correlated with personal achievement (Watts, 2014). Although moderate levels of anxiety can be beneficial for individuals in realizing their potential (Horwitz, 1996), the accumulation of anxiety among teachers can negatively affect their performance (Zhang et al., 2004). Chronic and excessive exposure to occupational stress can lead to significant mental and physical health issues. Currently, a growing body of research has focused on the impact of mental health on the teaching behaviors of primary and junior high school teachers, yielding a series of constructive findings. However, there is a scarcity of studies examining the internal relationships among workplace anxiety, job burnout, and self-efficacy from the perspective of teachers’ age. Notably, academic circles have paid relatively little attention to the mental health of young and middle-aged lecturers in universities. Promoting teachers’ mental health is crucial for ensuring teaching quality, maintaining harmonious teacher-student relationships, and enhancing teachers’ overall well-being. In the increasingly competitive environment of higher education, it is particularly urgent to understand how workplace anxiety influences job burnout among teachers. This understanding is essential for further exploring teachers’ mental health and optimizing the management strategies for young and middle-aged lecturers in universities.

## 2 Theoretical basis and research hypothesis

### 2.1 Career growth expectations influence workplace anxiety among young and middle-aged university lecturers

As an emotional experience that has existed in human society for a long time, anxiety is a significant area of study in modern psychology and medical research. Workplace anxiety, also referred to as occupational anxiety or work-related anxiety, can either impair or enhance job performance (Xu and Li, 2019). It has a dual effect, positively promoting or negatively impacting individual work performance. The intensity of anxiety is influenced by various factors, including individual characteristics such as environment, education level, and health status (Pan et al., 2022). Additionally, factors such as fear of negative evaluation (Powell et al., 2022), lack of social skills (Muschalla and Linden, 2013), and the dynamics of superior-subordinate relationships play a role in shaping anxiety levels (Wang et al., 2023). Moderate anxiety can stimulate individuals’ energy and foster creative behavior. However, excessive anxiety is a significant contributor to mental health issues, including depression and destructive behavior. Thus, anxiety possesses both a “dark side” and a “bright side,” which are related to job performance (Cheng and Mccarthy, 2018). As the prevalence of anxiety continues to rise, workplace anxiety has increasingly garnered the attention of academic researchers. Studies indicate that workplace anxiety not only affects individual behavior and emotions but also exhibits cross-situational characteristics, permeating into family life and disrupting familial relationships (Wang et al., 2018).

Research shows, “anxiety” and “expectation” are recognized as two interrelated variables. For instance, anxiety serves as a mediating factor between the expectation gap and investment in scientific research (Li and Xu, 2021), while family expectations positively influence college students’ anxiety levels (Zhang et al., 2022). Furthermore, research has indicated that anxiety indirectly contributes to job burnout (Fan et al., 2022). Teachers are among the groups with a high incidence of job burnout (Tian et al., 2022). With the emergence of the era of academic competition, higher education appears increasingly “prosperous” on the surface, yet remains “stagnant” in essence (Long, 2021). Studies focusing on primary and secondary school teachers have highlighted that excessive anxiety is not only a significant contributor to teachers’ job burnout (Jennett et al., 2003), but also poses a potential threat that indirectly affects students’ mental health (Zhou and Peng, 2004), which is significantly correlated with job burnout (Wu et al., 2015). However, there are significant differences in the relationship between efficacy and burnout among various groups of subjects (Chen and Tang, 2024). Young and middle-aged university lecturers are at a stage of professional development and are facing an increasingly complex pressure environment. Confronted with various challenges, including teaching reforms, research expectations, workplace competition, and social service obligations, they represent a significant group experiencing workplace anxiety. Consequently, is workplace anxiety a factor that enhances their work engagement, or is it a significant cause of their job burnout? Currently, there is a scarcity of relevant studies, and no definitive conclusions have been reached.

**Figure 1:**
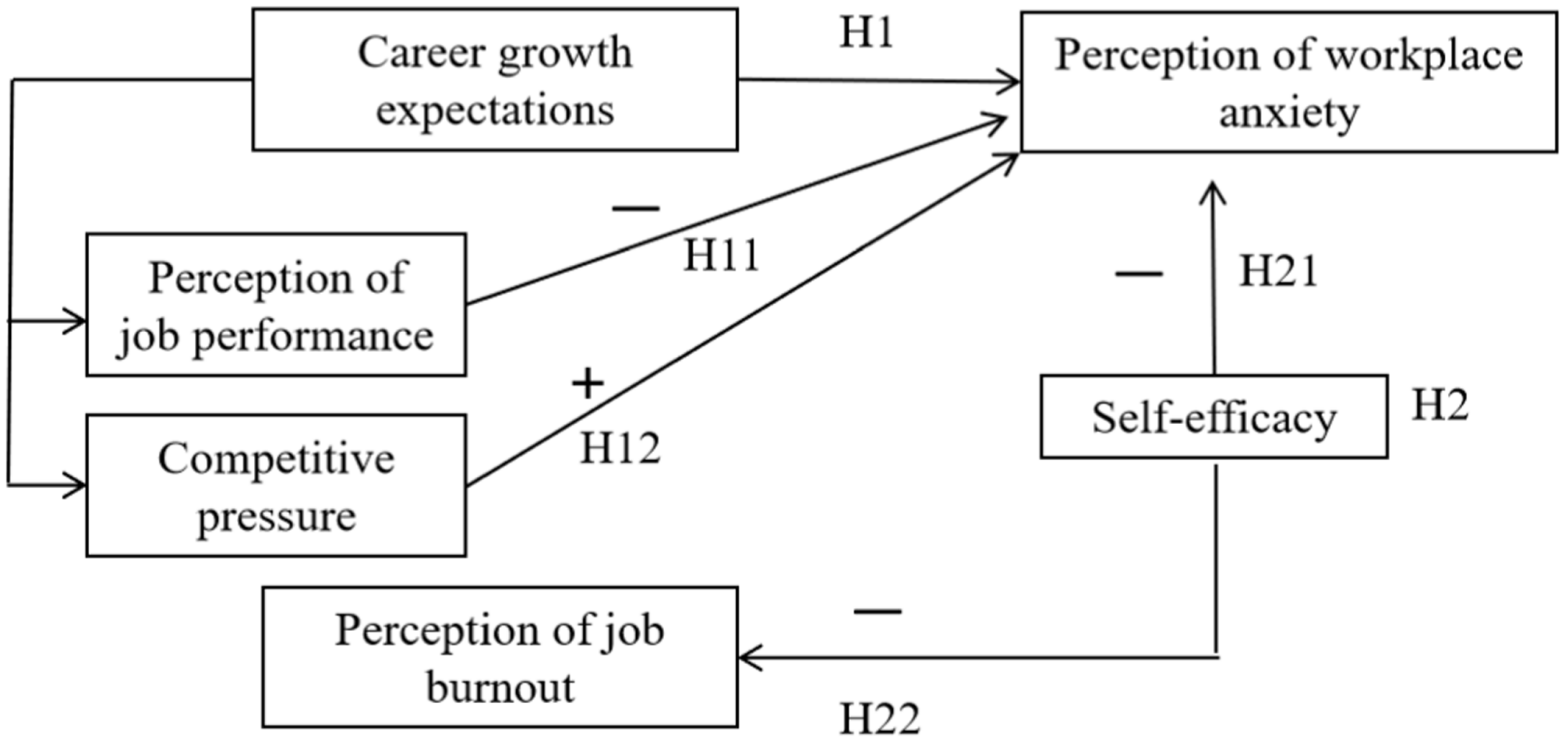
Assumed variables and their relationships.

Accordingly, this study focuses on young and middle-aged university lecturers as the primary research subjects. The following hypotheses are proposed (see Figure 1):

*H1*: The career growth expectations of young and middle-aged university lecturers significantly impact workplace anxiety. Specifically, higher career growth expectations correlate with increased levels of workplace anxiety; conversely, lower expectations are associated with reduced anxiety.

The following sub-assumptions are proposed:

*H11*: There is a negative correlation between job performance and the perception of workplace anxiety among young and middle-aged university lecturers. Specifically, as their sense of achievement in areas such as scientific research, teaching, social services, and professional title evaluation increases, their perception of workplace anxiety decreases. Conversely, a lower sense of achievement is associated with a higher perception of workplace anxiety.

*H12*: There is a positive correlation between competitive pressure and the perception of workplace anxiety among young and middle-aged university lecturers. Specifically, as the degree of involution in teaching, scientific research, social services, professional title evaluation, and other areas increases, so does their perception of workplace anxiety, and vice versa.

### 2.2 Self-efficacy plays an important regulatory role

Currently, a range of representative perspectives has emerged in the interdisciplinary research on workplace anxiety within the fields of psychology, education, and management. Paul et al. (2022) discovered in their study of Thai kindergarten, primary, and secondary school teachers that mental health is closely linked to job burnout. As an external manifestation of psychological exhaustion, job burnout results from the continuous accumulation of occupational anxiety and is characterized by negative emotional states such as emotional exhaustion, cynicism, and a sense of inefficacy (Maslach, 2003). This phenomenon is a significant barrier to the professional growth of teachers.

Existing research has examined the moderating factors of workplace anxiety from various perspectives and levels. For instance, fostering a sense of pride and accomplishment in one’s work (Gu et al., 2018), enhancing self-efficacy and self-confidence (Yang, 2009), and maintaining teachers’ perceptions of fairness in benefits all contribute to reducing workplace anxiety among teachers to varying extents (Meng and Bao, 2004). Notably, self-efficacy, as proposed by Bandura, is widely utilized as a theoretical framework to assess an individual’s confidence in completing specific tasks or goals (Bandura, 1977). In the educational context, teachers’ sense of efficacy has been recognized as a significant source of information that influences their teaching practices (Asih et al., 2022). It is also viewed as a self-awareness of teachers’ subjective evaluations of their ability to fulfill various job responsibilities (Cho and Shim, 2013).

Emotions and self-efficacy are interrelated psychological variables that significantly influence one another. Core self-evaluation, which encompasses self-efficacy, plays a crucial role in workplace anxiety (Xu and Li, 2019). Egyed and Short (2006) conducted research on American primary school teachers and found that teachers’ self-efficacy is inversely correlated with job burnou,while positively impacting students’ learning outcomes (Seçil et al., 2018). Hu et al. (2016) highlighted that self-efficacy serves as a mediating factor between teachers’ negative emotions and their job involvement. Nagase et al. (2020)examined Japanese primary school teachers and demonstrated that teachers’ emotional distress is significantly negatively correlated with their effectiveness in inclusive teaching, collaboration, and management behaviors.

Research has confirmed that factors such as social expectations, competitive mechanisms, and self-growth aspirations can contribute to varying degrees of workplace anxiety among teachers, which in turn can hinder their professional development and teaching effectiveness (Zhou, 2021). As the trend of involution intensifies within the university environment, job burnout among young and middle-aged teachers has also increased (Hu and Ren, 2022), further exacerbating workplace anxiety. Most existing studies have primarily focused on the workplace anxiety and coping strategies of kindergarten, primary, and secondary school teachers from the perspective of basic education. It remains unclear whether these conclusions are applicable to young and middle-aged university lecturers, and whether the relevant strategies and recommendations can effectively alleviate their workplace anxiety. Based on this context, the following hypotheses are proposed (see Figure 1):

*H2*: Self-efficacy plays a moderating role in alleviating workplace anxiety and job burnout among young and middle-aged university lecturers.

*H21*: There is a negative correlation between the self-efficacy of young and middle-aged university lecturers and their workplace anxiety.

*H22*: There is a negative correlation between the self-efficacy of young and middle-aged university lecturers and their job burnout.

## 3 Methodology

### 3.1 Sample selection

From September 2022 to October 2023, the research group conducted interviews with young and middle-aged lecturers from 10 universities in Chongqing, Anhui, Beijing, and Hebei using semi-structured interviews and online questionnaires. Throughout the investigation, we carefully considered the diversity of the research subjects in terms of professional background, subject area, and professional title requirements. The questionnaires were distributed through QQ groups, WeChat groups, Questionnaire Star, and other channels within universities, while the semi-structured interviews were conducted face-to-face. The research subjects included young and middle-aged lecturers in higher education who are involved in undergraduate or postgraduate instruction.

Based on the age classification standards set by the National Bureau of Statistics, and considering that young and middle-aged university lecturers typically have extensive educational backgrounds, this study defines the age range of young and middle-aged university lecturers as 25 to 55 years old. A total of 56 respondents were interviewed. The interview materials were utilized to validate the research conclusions and to propose targeted mitigation strategies. Additionally, 506 online questionnaires were collected, of which 449 were deemed valid after excluding incomplete, incorrect, and non-relevant responses, resulting in an effective response rate of 88.7%.

### 3.2 Questionnaire design

#### 3.2.1 Measurement factors and reliability analysis

To ensure the scientific rigor and logical coherence of the measurement items selected for the questionnaire, the design process drew upon established scales of workplace anxiety, job burnout, and self-efficacy utilized in existing research. The questionnaire includes independent variables, dependent variables, moderating variables, and control variables, selecting measurement dimensions and factors based on the characteristics of university lecturers. From the perspective of their subjective emotional experiences, workplace anxiety and job burnout were assessed. Due to the implicit nature of emotions such as anxiety and burnout, the questionnaire was designed to measure items from easily perceptible angles such as “physical condition,” “mental stress,” “perception of workplace competition,” and “sense of work achievement.” This led to the identification of four measurement dimensions, aside from the control variable “personality traits” (Table 1). The questionnaire comprised a total of 24 items, including 4 personal information questions and 20 items designed to evaluate respondents’ levels of workplace anxiety, job burnout, and self-efficacy. Responses were measured using a five-point Likert scale, ranging from “strongly agree” to “strongly disagree,” corresponding scores of 1 to 5. Notably, a higher score indicates a lower level of agreement. The adjusted standardized Cronbach’s *α* coefficient for the questionnaire exceeded 0.7, demonstrating good reliability (see Table 2).

**Table 1:** Dimensions and Factors of Measurement Items.

Variable properties	Questionnaire measurement dimension	Measurement factor
Independent Variable	A. Career growth expectations	a1 Professional Expectations, a2 Performance Expectations, a3 Reputation Expectations, a4 Others Expectations
Dependent Variable	B. Perception of workplace anxiety	b1 Stress Coping, b2 Physical Exhaustion, b3 Workplace Competition, b4 Confidence Perception, b5 Competition Internal Volume
	C. Perception of job performance	c1 Leader Recognized c2 Family Recognized c3 Research Performance
	D. Perception of Job burnout	d1 Mental Exhaustion, d2 Struggle, d3 Dehumanization, d4 Frustration
Regulating Variable	E. Self-efficacy	e1 Ability Recognized, e2 Experience Recognized, e3 Resilience, e4 Emotional Arousal
Control Variable	F. Personality traits	f1 Age, f2 Education Level, f3 Subject Background, f4 Title Status

**Table 2:** Reliability of the questionnaire.

	Cronbach’s α	Standardize Cronbach’s *α*	Number of terms	Sample size
Before the adjustment	0.616	0.648	20	449

	Cronbach’s α	Standardize Cronbach’s α	Number of terms	Sample size
After the adjustment	0.701	0.738	15	449

Remove the options with a negative population relationship (b2, d4, c2, c3, e4) to obtain the adjusted α.

#### 3.2.2 Variable interpretation

##### 3.2.2.1 Independent variables

According to the previous research and the accompanying interview data, the independent variable is defined as “career growth expectation” (Huang et al., 2022). Variable is assessed by gaging the respondents’ perceptions of their expectations in scientific research, teaching, and professional advancement. The questionnaire was designed with four topics to collectively elucidate the individual performance of respondents across the four variables (a1-a4) as shown in Table 1. A five-point Likert scale was employed to evaluate the respondents’ career growth expectations. The results indicate that the mean value of this data segment is 1.99, with a frequency exceeding 50%, and the median is 2 (refer to Table 3). This suggests that the majority of the interviewed teachers maintain a positive attitude toward the four dimensions of career growth expectations (a1, a2, a3, and a4).

**Table 3:** Descriptive statistics of main variables.

Variable type	Variable dimension	Measurement factor	Correlation hypothesis	Frequency accumulation over 50% option	Maximum value	Minimum value	Mean value	Median
Core argument	A. Career growth expectations	a1,a2, a3,a4	Career growth expectations → Workplace anxiety	agree	5	1	1.99	2
Dependent variable	B. Perception of workplace anxiety C. Job performance perception D. Job burnout perception	b1,b3,b4,c1,c3,c4,d1,d2,d3	Workplace anxiety → Job burnout	Agree, not necessarily	5	1	2.20	2
Regulating variable	E.Self-efficacy	e1, e2, e3	Workplace anxiety → self-efficacy → Job burnout	Agree	5	1	1.89	2
Control variable	F. Control characteristics	f1	——	[31–40]	3	1	1.78	2
		f2		PhD	2	1	1.63	2
		f3		Humanities and Social Sciences	2	1	1.45	1
		f4		Lecturer or an equivalent title, Associate Professor or an equivalent title	4	1	2.37	2

The “→” between two variables indicates that the two variables are assumed to be correlated. The “→” symbol connecting three variables suggests that the intermediate variable has a moderating effect on the left and right variables. Additionally, the data are presented with two decimal places, as will be the case in the following sections.

##### 3.2.2.2 Dependent variables

Based on the research findings of Li and Shi (2003) and Aleksandra et al. (2005), along with interview data, the dependent variable of this study (i.e., the explained variable) comprises three dimensions: workplace anxiety perception, job performance perception, and job burnout perception. It also includes nine measurement factors, such as stress coping (b1) and workplace competition (b3) (see Table 1). According to the descriptive statistics presented in Table 3, the median of the nine items is 2, while the average value is 2.2. This indicates that more than half of the respondents selected the “agree” and “very agree” options, suggesting that the respondents generally exhibit a positive attitude toward workplace anxiety perception, job performance perception, and job burnout perception.They perceive varying levels of workplace anxiety.

##### 3.2.2.3 Regulating variable

Combined with the interview data, this study examines whether self-efficacy moderates the relationship between workplace anxiety and job burnout among young and middle-aged university lecturers. The questionnaire assesses moderating variables, which include ability recognized (e1), experience recognized (e2), and resilience (e3). A total of three items were designed to evaluate respondents’ perceptions of their personal abilities, historical experiences, and resilience, as well as the extent to which these factors can either mitigate or exacerbate their workplace anxiety. The descriptive results reveal that the median score for the moderating variable is 2, while the average score is 1.89, indicating that more than 50% of respondents selected the and ‘agree ‘options.

##### 3.2.2.4 Control variables

According to the measurement table provided by Muhammad (2022), and considering the overall situation of young and middle-aged university lecturers, the control variables consist of the personality characteristics of the respondents. These include age (f1), education level (f2), subject background (f3), and professional title status (f4). Notably, more than half of the respondents are aged between 31 and 40 years. This age group represents the backbone of discipline construction and development within colleges and universities, forming the majority of young and middle-aged lecturers. Most respondents hold a master’s degree or higher, with the majority holding professional titles of lecturer or associate professor. They are currently in a phase of professional advancement and academic growth, which aligns with the objectives of this study.

## 4 Quantitativedata analysis

### 4.1 Discriminant validity analysis

After removing unreasonable items (b2, d4, c2, c3, and e4) based on reliability analysis, the average values were utilized to generate and merge relevant variables. The confirmatory factor analysis method was then employed to assess model fit.*p*-value, GFI, RMSEA, RMR, CFI, NFI and NNFI are key indicators used to evaluate the superiority of model fit when conducting a discriminative validity test with SPSS software. Among these, the p-value serves as a measure of statistical significance. If the p-value is less than the predetermined significance level (typically set at 0.05), it indicates that the correlation between the two variables is statistically significant, suggesting a degree of relationship that may imply insufficient discriminative validity. In addition, the Goodness of Fit Index (GFI), Comparative Fit Index (CFI), Normed Fit Index (NFI), and Non-normed Fit Index (NNFI) serve as positive indicators for assessing the goodness of fit of a model. These values range from 0 and 1. The closer the value is to 1, the better the model fits. Generally, when the values of the indicators mentioned above exceed 0.9, it indicates a superior model fit. Conversely, the Root Mean Square Error of Approximation (RMSEA) and the Root Mean Square Residual (RMR) serve as opposing indicators, as they account for the complexity of the model. The closer the values of RMSEA and RMR are to 0 (typically RMSEA <0.1 and RMR < 0.05), the better the model’s fit. Thus, the value ranges of these indicators provide a reliable framework for a comprehensive evaluation of the model’s fit.

As presented in Table 4, the primary evaluation indices for the five-factor model are as follows: GFI = 1.68 (> 0.9), RMSEA = 0.04 (< 0.10), RMR = 0.033 (< 0.05), and CFI = 0.997 (> 0.9). All these indices meet the corresponding reference standards. This indicates that the five-factor model provides the best fit, and the main factors selected in this study demonstrate good discriminant validity.

**Table 4:** Comparison of model fit degree.

Model	*X* ^2^	df	*p*	Chi-square degrees of freedom ratio	GFI	RMSEA	RMR	CFI	NFI	NNFI
Standard of reference	–	–	>0.05	<3	>0.9	<0.10	<0.05	>0.9	>0.9	>0.9
Five factors (1, 2, 3, 4, 5)	777.95	80	0.122	2.72	1.68	0.04	0.033	0.997	0.977	0.902
Four factors (1, 2, 3, 4, 5)	904.55	84	0.068	5.77	0.92	0.148	0.092	0.643	0.624	0.554
Three factors (1, 2, 3, 4, 5)	406.49	74	0.056	5.49	0.52	0.1	0.08	0.561	0.521	0.461
Two factors (1, 2, 3, 4, 5)	1025.46	89	0.045	11.52	0.57	0.153	0.102	0.593	0.574	0.52
Single factor (1 + 2 + 3 + 4 + 5)	1132.83	90	0	12.59	0.53	0.161	0.097	0.547	0.529	0.471

The test factors include career growth expectation (1), workplace anxiety perception (2), job performance perception (3), job burnout perception (4) and self-efficacy (5).

**Table 5:** Correlation between involution competition and career growth expectations.

	b5 Competition internal volume	a2 Performance expectations	a3 Reputation expectations	a4 Others expectations
b5	1 (0.000***)	0.162 (0.001***)	0.125 (0.008***)	0.087 (0.067*)
a2		1 (0.000***)	0.448 (0.000***)	0.191 (0.000***)
a3			1 (0.000***)	0.397 (0.000***)
a4				1 (0.000***)

***, **, and * represent significance levels of 1, 5, and 10%, respectively, as indicated above.

### 4.2 Hypothesis testing and process analysis

After generating variables and combining measurement factors, the Spearman correlation coefficient method was employed to analyze the correlation between the primary variables. The analysis revealed that the career growth expectations of young and middle-aged university lecturers exhibited the following characteristics in relation to their psychological emotions, such as workplace anxiety and job burnout:

1. Career growth expectations have a positive impact on workplace anxiety (*r* = 0.208***), thus validating Hypothesis H1.
2. A strong negative correlation exists between perceived workplace anxiety and job performance (*r* = −0.36***), confirming Hypothesis H11, indicating a significant inverse relationship between these two variables (Table 6).
3. A weak positive correlation was found between the introspection of university competition and teachers’ career growth expectations (*r* = 0.162***, 0.125***, and 0.087***), supporting Hypothesis H12 (Table 5). This correlation can be attributed to the peer effect in workplace competition, suggesting that the development of introspection in the university environment has a reinforcing effect on promoting teachers’ upward motivation.
4. Consistent with findings from existing studies, a strong negative correlation was identified between self-efficacy and the perception of workplace anxiety among young and middle-aged university lecturers (*r* = −0.36***), thereby establishing Hypothesis H21. Additionally, a positive correlation was observed between perceived job burnout and career growth expectations (*r* = 0.188***; Table 6). This indicates that as respondents recognize their career growth expectations, their sense of job burnout may also increase, potentially reflecting a negative effect stemming from the dual influences of workplace anxiety and career stress. However, there is no direct correlation between self-efficacy and job burnout for young and middle-aged university lecturers, rendering Hypothesis H22 invalid. At this point, Hypotheses H11, H12, H21, and H22 have all been evaluated.

**Table 6:** Correlation among the five variables.

	E. Self-efficacy	B. Perception of workplace anxiety	C. Job performance perception	D. Job burnout perception	A. Career growth expectation
E	1 (0.000***)	−0.36 (0.000***)	0.101 (0.032**)	−0.041 (0.384)	0.437 (0.000***)
B		1 (0.000***)	−0.36 (0.000***)	0.185 (0.000***)	0.208 (0.000***)
C			1 (0.000***)	0.323 (0.000***)	0.135 (0.004***)
D				1 (0.000***)	0.188 (0.000***)
A					1 (0.000***)

### 4.3 Moderating effect and test analysis

To further investigate whether self-efficacy moderates the relationship between workplace anxiety and job burnout among young and middle-aged university lecturers (H2), we employed the SPSS moderation analysis and utilized a Bootstrap sampling method with 1,000 iterations. We successively tested the relationships among workplace anxiety perception (independent variable B), job burnout (dependent variable D), and self-efficacy (moderator variable E).In general, the test results of the SPSS model indicate that a higher value of the variable R^2^ or ΔR^2^ corresponds to a better fit of the model. The results of Model 3 indicate that the adjusted ▵*R*^2^ is 0.087, which is an improvement over Model 1 (▵*R*^2^ = 0.055) and Model 2 (▵*R*^2^ = 0.068) while the interaction term between “workplace anxiety perception” and the significance *p*-value is 0.001***. This demonstrates that the moderating variables significantly impact the original model, and Model 3 is crucial in explaining the variations in the dependent variables. This suggests that self-efficacy significantly influences the relationship between workplace anxiety perception and job burnout perception among young and middle-aged university lecturers (see Table 7).

**Table 7:** The moderating effect of self-efficacy on workplace anxiety and job burnout.

	Model 1				Model 2				Model 3			
	Coefficient	Standard error	*t*	*p*	Coefficient	Standard error	*t*	*p*	Coefficient	Standard error	*t*	*p*
Const	2.02	0.103	19.57	0.000***	1.77	0.14	12.82	0.000***	2.69	0.31	8.60	0.000***
B	0.24	0.045	5.21	0.000***	0.14	0.05	2.65	0.008***	−0.144	0.12	−1.17	0.24
E					0.23	0.05	5.10	0.000***	−0.335	0.15	−2.17	0.030**
B*E									0.19	0.06	3.25	0.001***
*R* ^2^	0.057				0.072				0.093			
Adjust *R*^2^	0.055				0.068				0.087			
*F*	*F*(449, 1) = 27.129, *p* = 0.000***				*F*(2, 446) = 17.27, P = 0.000***				*F*(3,445) = 15.276, *p* = 0.000***			
▵*R*^2^	0.057				0.072				0.093			
▵*F*	▵*F*(1, 449) = 27.13, *p* = 0.000***				▵*F*(1, 446) = 7.04, *p* = 0.001***				▵*F*(1, 445) = 28.19, *p* = 0.000***			

D, Dependent variable.

## 5 Qualitative results

As a negative emotion, workplace anxiety has become a prevalent psychological condition among young and middle-aged university lecturers. Its detrimental effects significantly impact teachers’ physical and mental health, as well as their professional development. This study sampled 449 young and middle-aged university lecturers and utilized self-efficacy as a moderating factor to analyze the interaction and mechanisms between workplace anxiety and job burnout. Through a questionnaire survey and empirical analysis, the research findings indicate that:

Firstly, in line with existing research, the career growth expectations of young and middle-aged university lecturers significantly impact their workplace anxiety (H1). Additionally, there is a negative correlation between the perception of job performance and workplace anxiety among university lecturers (H11). As the interviewee, FXM (42 years old, female), mentioned: *“My workplace anxiety primarily stems from my application for the position of associate professor. Currently, I am a lecturer with only a master’s degree. The increasing proportion of PhD in academic institutions adds to my pressure when evaluating faculty titles.” It is evident that competitive pressure positively correlates with the perception of workplace anxiety among young and middle-aged university lecturers (H12).* Furthermore,peer competition in the context of reinvention significantly exacerbates their workplace anxiety. The greater the expectations teachers have regarding professional titles, salary, social reputation, and recognition from others, the stronger their sense of anxiety becomes.

Secondly, self-efficacy, as a significant factor in emotion regulation, positively influences the reduction of professional anxiety among young and middle-aged university lecturers. Interviewee YHQ (35 years old, male) stated: *“I graduated from one of the top universities in China and earned a PhD degree. I have published 12 articles that have been indexed by SCI. I am confident that I can achieve the title of Assistant Professor within the next 3 years. I do not feel overly pressured by my current teaching and research commitments.”* When interviewees demonstrate a strong sense of self-efficacy in their teaching abilities, research capabilities, work experience, and adaptability, their perception of workplace anxiety tends to be correspondingly low (validating Hypothesis H21). Furthermore, the Bootstrap sampling results indicate that the self-efficacy of young and middle-aged university lecturers significantly influences the relationship between their perception of workplace anxiety and their experience of job burnout (as illustrated in Table 7). This suggests that self-efficacy plays a moderating role in alleviating workplace anxiety and job burnout among young and middle-aged university lecturers (validating Hypothesis H2).

In addition, research on police officers, medical workers, middle school teachers, and other groups has demonstrated that self-efficacy has a significant moderating or direct effect on job burnout within these populations (Shen and Zheng, 2005; Yao et al., 2013; Wang et al., 2007). However, these conclusions are not universally applicable. The data above shows that H22 was rejected, suggesting that there is no negative correlation between self-efficacy and job burnout. Furthermore, from a psychological perspective, H22 was invalid may indicate that the interviewees has high emotional intelligence. Therefore, when individuals experience a decrease in self-efficacy, they can better understand and regulate their emotions. This understanding fosters positive thinking and emotional development, thereby mitigating the risk of job burnout.

## 6 Conclusion and implications for future research

The group of young and middle-aged university lecturers is growing increasingly large. They not only bear the significant responsibility of teaching and educating individuals but also serve as a crucial force in promoting the high-quality development of higher education. Reducing workplace anxiety in professional development is a necessary prerequisite for ensuring the physical and mental well-being of young and middle-aged university lecturers. Furthermore, it is a key factor in preventing job burnout and enhancing professional satisfaction.

### 6.1 Construction of a positive working atmosphere: emphasizing humanistic care in the workplace and enhancing teachers’ emotional intelligence

The enhancement of school climate, particularly in terms of teacher-student relationships and teaching innovation, not only alleviates teachers’ anxiety but also improves their self-efficacy (Jiang et al., 2022). In China, university lecturers experience a markedly different professional growth environment compared to primary and secondary school teachers. First and foremost, young and middle-aged teachers are often the primary earners in their families. They bear the dual responsibility of raising children and caring for elderly relatives, while also needing to enhance their personal competitiveness through further education. This often results in significant economic and academic pressure. Additionally, unlike their counterparts in primary and secondary education, young and middle-aged university lecturers encounter a more competitive incentive system. Consequently, it is particularly urgent to create a respectful, understanding, and supportive work environment to help them maintain their mental health. From a psychological counseling perspective, colleges and universities should establish a workplace care mechanism tailored to the characteristics of young and middle-aged lecturers, as well as develop a standardized supervision and counseling pathway. By utilizing discussions, online communication, expert consultations, and other methods, institutions can gain timely insights into teachers’ mental health status and foster a sense of belonging and community among educators. Research indicates that emotional intelligence negatively correlates with anxiety perception. Therefore, to strengthen emotional intelligence, schools should actively engage teachers in enhancing their self-awareness of emotions. This involves guiding teachers to recognize anxiety and its potential hazards promptly, establishing regular stress relief channels through union activities, collaborative teaching and research, individual support, sports, and other initiatives. Additionally, schools should promote self-assessment methods for workplace anxiety and strategies for managing workplace burnout. These efforts aim to help teachers mitigate or reduce the negative impacts of anxiety while gradually improving their emotional intelligence.

### 6.2 Reforming the competition mechanism: preventing the escalation of involution and enhancing teachers’ professional efficacy

Colleges and universities serve as hubs for experts, scholars, and educational leaders, representing institutions of higher education, knowledge, and intelligence. As interviewee ZYL (36, female) mentioned: *“I missed the optimal time to apply for assistant professor positions. Based on my current achievements, I have fulfilled all the basic requirements for a senior title. However, my colleagues all hold PhDs, and the competition among peers is getting fiercer.”* Chinese universities have implemented increasingly stringent requirements for faculty, which are evident in the expectations for academic qualifications, research contributions, and teaching experience. As a result, competition among university lecturers have become increasingly intense.

Interview data indicate that, in light of increasingly stringent job requirements, professional title evaluations, and peer competition, young and middle-aged university faculty members in the early stages of their careers are experiencing negative psychological phenomena. These include led to doubts regarding their self-efficacy and, in some cases, a descent into a “opt-out” professional development. The underlying causes of these issues can be attributed to two primary factors: the external environment and internal mechanisms. On one hand, the overall qualifications of faculty members have been rising, particularly with the influx of numerous young PhD graduates into academia in recent years. This trend has intensified competitive pressure on middle-aged lecturers who possess lower academic credentials and professional titles. On the other hand, there is a disconnect between the traditional mechanisms for professional growth within universities and the current demands for faculty advancement.

As the opposite of “inwardness” leading to “opt-out,” the humanized reform of the competition mechanism is more inspiring and scientific to improve the professional efficacy of young and middle-aged university lecturers. Therefore, in alignment with the expectations for high-quality development in higher education, colleges and universities should integrate various aspects of the teacher assessment mechanism, including the quality of education and teaching, the prospects for discipline development, and the evaluation of teacher capabilities. This approach should establish a growth pathway tailored to different types of teachers—those engaged in teaching, teaching research, scientific research, and social service—broadening the avenues for professional development and encouraging the diverse enhancement of teachers’ skills. Additionally, it is essential to improve the scientific rigor and credibility of the professional title evaluation system. By creating a more attractive talent system externally, we can reduce teachers’ occupational anxiety, prevent job burnout, and enhance professional efficacy internally.

### 6.3 Research limitations and future prospects

Based on the technical route of “hypothesis testing-questionnaire sampling-data analysis,” this study conducted an empirical analysis of the relationship between lecturers’ workplace anxiety, job burnout and self-efficacy. This study collected data from 449 young and middle-aged university lecturers, establishing a reliable sample. The paper employed a combination of SPSS quantitative methods and semi-structured qualitative interviews to conduct an empirical analysis of the relationship between workplace anxiety and job burnout among lecturers. This research provides valuable insights into workplace anxiety and job burnout experienced by university professors. The findings contribute to the understanding of mental health issues faced by young and middle-aged lecturers in universities.

However, this paper has several research limitations. For instance, not all relevant variables are considered due to constraints related to length, subject matter, and research methodology. Specifically, the potential influence of external factors, such as family environment and other social dynamics, on lectures’ workplace anxiety and job burnout is not addressed. Additionally, while the questionnaire options have been technically refined, respondents may still choose to avoid or downplay certain questions due to various psychological concerns. Furthermore, the research samples are primarily drawn from Beijing, Hebei, Chongqing, and Anhui provinces in China, which may restrict the generalizability of the findings to other countries. Future studies could benefit from including a broader range of young and middle-aged university lecturers from diverse regions, allowing for multi-dimensional and multi-method analyses of the negative impacts of workplace anxiety perception. This could involve further refining the strength scale of workplace anxiety perception and examining the personality traits of respondents, as well as employing a combination of qualitative research and empirical testing to gain deeper insights.

## Data Availability

The original contributions presented in the study are included in the article/supplementary material, further inquiries can be directed to the corresponding author/s.
